# Chromosome-level genome assembly for the ecologically and economically important alga *Saccharina japonica*

**DOI:** 10.1038/s41597-025-04620-w

**Published:** 2025-02-18

**Authors:** Xiaodong Li, Yu-Long Li, Chenhui Zhong, Jing Li, Li Su, Jin-Xian Liu, Shaojun Pang

**Affiliations:** 1 Key Laboratory of Breeding Biotechnology and Sustainable Aquaculture (CAS), Institute of Oceanology, Chinese Academy of Sciences, Qingdao, China; 2 Laboratory for Marine Biology and Biotechnology, Qingdao Marine Science and Technology Center, Qingdao, China; 3 CAS Key Laboratory of Marine Ecology and Environmental Sciences, Institute of Oceanology, Chinese Academy of Sciences, Qingdao, China; 4 Laboratory for Marine Ecology and Environmental Science, Qingdao Marine Science and Technology Center, Qingdao, China; 5 Provincial Key Laboratory of Cultivation and High–value Utilization of Marine Organisms, Fisheries Research Institute of Fujian Province, Xiamen, China

**Keywords:** Genome, Agricultural genetics

## Abstract

*Saccharina japonica* is a major kelp species of brown algae with the highest production among aquaculture seaweeds and holds important ecological and economic value. Despite advancements in domestication, a high-quality chromosome-level genome assembly is needed to assist its genetic improvement. Previous genome assemblies of *S. japonica* were either on a draft-level or highly fragmented. Here, we generated a high-quality chromosome-level genome for the female sporophyte using PacBio sequencing and Hi-C. The genome is 516.11 Mb, with contig N50 length of 491.30 Kb and scaffold N50 length of 16.24 Mb, anchored into 32 pseudo-chromosomes. Repetitive elements constituted 45.07% of the genome, and 17,739 protein-coding genes were predicted, of which 82% were functionally annotated. This genome provides a crucial resource for biotechnological advances in *S. japonica* breeding and offers insights into the ecology and evolution of brown algae.

## Background & Summary

The brown macroalga *Saccharina japonica* is a major component of intertidal seaweed flora in the northwest coast of the Pacific Ocean, including Russia, Japan, Korea and China^1^. It is one of the most economically important seaweeds in aquaculture, being extensively utilized as human food, marine animal feeds and raw industrial materials for various end products^2^. Through artificial cultivation, the output of *S. japonica* reached 10.86 million tons in fresh weight and had a market value of more than 4.4 billion US dollars in 2020^3^. Over the past decades, this species has become one of the most cultivated macroalgae in China^4^. Using traditional selective breeding and/or hybridization techniques, more than a dozen certified varieties have been successfully bred and farmed in China, which have contributed to the farming success of this cold-water species in both the temperate and subtropical regions along the coasts of China.

High quality genomic information can provide important resources for biotechnological approaches to crop genetic improvements. Despite recent advances in molecular-aided genetic breeding, genetic structure assessments and transcriptomic studies in this important marine farmed “crop”^4–10^, *S. japonica* remains to be a species with limited genetic engineering tools due to the absence of a high-quality genome assembly. By using Illumina reads and 10 Gb of PacBio long reads, Ye *et al*.^11^ reported a draft genome of *S. japonica* with assembly size of 537 Mb and contig N50 length of 58.87 Kb. By using the High-throughput Chromosome Conformation Capture (Hi-C) data and Illumina reads, Liu *et al*.^12^ assembled a chromosome-level genome of *S. japonica* with genome size of 580.5 Mb and contig N50 length of 4.73 Kb. However, the genome assembly is fragmented and annotations reported in Liu *et al*.^12^ are not publicly accessible. Considering the ecological and economic importance of *S. japonica*, a high-quality chromosome level genome assembly is still urgently needed.

In this study, we generated a chromosome-level genome assembly (516.11 Mb) of *S. japonica* female sporophyte using Illumina, PacBio HiFi, and Hi-C data. The assembly exhibited a contig N50 length of 491.30 Kb and scaffold N50 length of 16.24 Mb, indicating superior continuity compared to the previously reported ones. Approximately ~96.15% of the genome was anchored to 32 chromosomes, consistent with the chromosome number reported for species previously classified in the Laminaria genus, including *Saccharina japonica*^13^. Repetitive elements constituted 45.07% of the genome assembly. The evaluation indexes of the new assembly such as the Merqury QV (34.86), the completeness (96.67%) and the mapping ratio of pair-end short reads (87.16%) indicate a higher level of genome completeness. A total of 17,739 protein-coding genes were predicted and functional annotations were assigned to 14,510 (82%) of them, much enhanced the interpretative power.

This high-quality chromosome‐level *S. japonica* genome, overcoming the limitations of the previous versions, provides a foundational genomic resource for developing molecular approaches in future breeding efforts and offers insights for ecological and evolutionary studies of the brown algae in general.

## Methods

### Preparation of biological materials

#### Gametophyte source and gametogenesis condition

The female gametophyte strain HJS-C used in this study was zoospore-derived clone preserved in Seaweed Culture Collection Center, Chinese Academy of Sciences (http://www.caslivealgae.com). The strain was stored in liquid culture medium in a glass tube in a temperature-controlled incubator at 10 °C under 10 μmol photons m^−2^ s^−1^ white light with a 12 h: 12 h light-dark cycles.

For gametogenesis, the filamentous gametophytes were broken down into 5–10 cell fragments using an electric blender (20, 000 r/min. JYL-C012, Jiuyang, China). The fragments were inoculated into 9 cm Petri dishes at an average density of 10–15 fragments per microscopic field (100 × magnification). Gametogenesis was carried out at 12 °C, 50 μmol photons m^−2^ s^−1^ in white light in PES^14^ with a medium renewal period of three days and a photoperiod of 12:12 (L: D).

#### Culture of the female sporophyte

When parthenogenetic sporophytes, formed through haploid doubling, appeared in the HJS-C culture petri dishes after 20 day’s culture, they were subsequently transferred into a 1-l glass beaker containing PES medium for further culture in aeration using compressed air. The culture beaker was placed in a temperature and light-controlled incubator at 12 °C in 60 μmol photons m^−2^ s^−1^ white light with a photoperiod of 12:12 (L: D). As the sporophytes reached 3–5 cm in length, they were transferred into an 80-l flow-through tank for grown-up. The tank was set in a room with reduced ambient solar irradiance. Daily PAR went up to maximally 300 μmol photons m^−2^ s^−1^ for ca 2 h on a sunny day. The sporophytes were tumbled in the water currents as described in Pang *et al*.^15^. Daily water temperature varied between 12 to 14 °C. Every two days, KNO_3_ and KH_2_PO_4_ were added to reach levels of 10 and 1 mg L^−1^, respectively.

The algal material used for genome sequencing has been preserved in the Seaweed Culture Collection Center, Chinese Academy of Sciences (http://www.caslivealgae.com) and is accessible for research purposes according to standard repository procedures.

#### Sample preparation and genomic DNA extraction

Female sporophytes were harvested when they reached 10 cm in length. To ensure the least amount of contamination during PacBio sequencing, the sporophytes were repeatedly treated with antibiotics until no bacterial colonies would form when plated. For antibiotic treatments, a stock solution of gentamicin was prepared at a final concentration of 200 µg mL^−1^. The pre-washed sporophytes were immersed in this solution for a duration of 2 hours. Following this treatment, the sporophytes were vigorously shaken in autoclaved seawater to dislodge surface bacteria that had been inhibited or killed. Bacteria were determined by plate coating method. For sampling, sporophytes were rapidly frozen with liquid nitrogen, and subsequently preserved at −80 °C. Genomic DNA was extracted from the female sporophyte using improved CTAB method.

### Library preparation and sequencing

In brief, SMRTbell libraries were constructed for the long read sequencing based on PacBio’s standard protocol (Pacific Biosciences, Menlo Park, CA, USA). These libraries were sequenced through a PacBio Sequel II System (Pacific Biosciences). About 2.36 million HiFi reads (39.55 Gb) with a mean length of 16,763 Kb were generated. For the Hi-C sequencing, genomic DNAs were fixed with formaldehyde, sheared by the restriction enzyme MboI to build a Hi-C library, and then sequenced on a NovaSeq6000 sequencing platform (Illumina Inc., San Diego, CA, USA). A total of 53.19 Gb of 150 bp paired-end Hi-C data were generated (Table 1).

**Table 1:** Statistics of *S. japonica* genome assembly and annotations in this study.

Items	Statistical result
**Sequencing**	
Short reads sequencing	
Raw data (Gb)	58.14
Sequencing depth (X)	104
PacBio sequencing	
Raw data (Gb)	39.55
Sequencing depth (X)	71
Hi-C sequencing	
Raw data (Gb)	53.19
Sequencing depth (X)	95
**Assembly features**	
PacBio sequencing assembly	
Genome size (Mb)	516.11
Number of contigs	2802
Contig N50 (Kb)	491.30
Hi-C assembly	
Genome size (Mb)	516.35
Chromosome number	32
Anchored rate (%)	96.15
Number of scaffolds	405
Scaffold N50 (Mb)	16.24
**Genome annotation**	
Number of protein-coding genes	17739
Number of genes annotated	14510
Average gene length (bp)	12489
Average exon length (bp)	231
Average exons per gene	6.99
Number of exons	123996
Average intron length (bp)	1816
Average introns per gene	5.99
Number of introns	106257
Total size of TEs (Mb)	215.21
TEs in genome (%)	41.76
Total size of repeated sequences (Mb)	232.3
Repeated sequences (%)	45.07

### Genome assembly

**Fig. 1:**
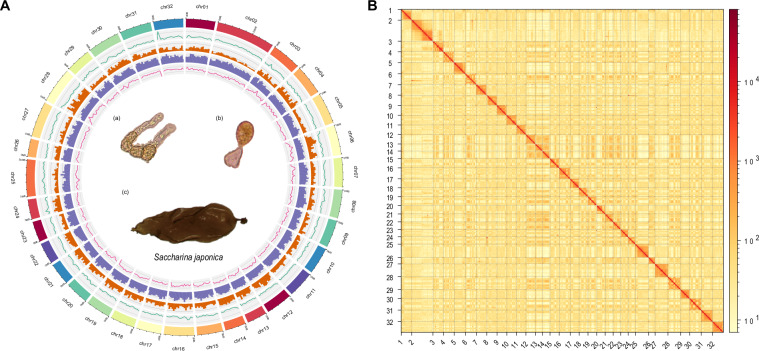
Chromosome-level genome assembly of *S. japonica*. (**A**) Circos plot of the *S. japonica* genome assembly. From outer to inner layers were chromosomes, depth of short-reads, gene densities, repetitive elements densities and GC contents, respectively. (a,b) female gametophyte, (c) female sporophyte. (**B**) Hi-C contact map for the genome assembly of *S. japonica*.

To assemble the genome, hifiasm (v0.19.9-r616)^16^ was employed to generate contigs utilizing PacBio HiFi reads using parameters “–hg-size 550 m -l 0”. Contigs sequences were checked for adaptors and contaminants using the NCBI Foreign Contamination Screen (FCS) tools (https://github.com/ncbi/fcs) and BlobTools2^17^. After removing potential adaptors and contaminations, contigs were further polished using high-quality short reads with NextPolish2^18^. Subsequently, the assembled contigs were organized into chromosomes utilizing the allhic v0.9.8^19^, followed by manual curation with JuiceBox (v2.17)^20^. To further improve the continuity, the scaffolds were gap-filled with HiFi reads using TGS-GapCloser 1.2.1^21^. The final assembly comprised 32 chromosomes (Fig. 1), collectively spanning 496.47 Mb, accounting for approximately 96.15% of the entire genome assembly, while the individual chromosome lengths ranged from 32.51 Mb (Chr2) to 9.90 Mb (Chr26) (Table 2). Comparing with the previous genome assembly of *S. japonica* presented by Ye *et al*.^11^ and Liu *et al*.^12^, our assembly has much longer contig and scaffold N50 (Table 3), which indicated the high-quality of our assembly, with superior continuity.

**Table 2:** Statistics of chromosome length in *S. japonica*.

Chromosome ID	No. contigs	Length (bp)	Percentage (%)
1	62	16,237,944	3.14
2	43	32,514,357	6.30
3	80	16,341,770	3.16
4	107	16,504,999	3.20
5	51	17,304,895	3.35
6	107	16,664,931	3.23
7	65	16,247,666	3.15
8	70	15,757,540	3.05
9	61	15,140,275	2.93
10	67	14,870,419	2.88
11	72	14,602,799	2.83
12	106	14,590,997	2.83
13	96	11,013,574	2.13
14	93	11,528,183	2.23
15	77	14,259,657	2.76
16	86	16,910,554	3.28
17	58	13,768,380	2.67
18	85	13,553,254	2.62
19	95	13,627,293	2.64
20	59	12,995,717	2.52
21	87	12,205,725	2.36
22	81	11,781,832	2.28
23	52	11,346,182	2.20
24	75	11,081,855	2.15
25	53	20,615,488	3.99
26	62	9,896,441	1.92
27	49	19,730,202	3.82
28	134	19,736,508	3.82
29	48	13,869,707	2.69
30	87	17,721,060	3.43
31	76	17,475,613	3.38
32	62	16,569,579	3.21
Total	2406	496,465,396	96.15

**Table 3:** Statistics of *S. japonica* genome assembly and annotations.

Statistics	*S. japonica* (This study)	*S. japonica* (Liu *et al*.^12^)	*S. japonica* (Ye *et al*.^11^)
Platform	Illumina + PacBio +Hi-C	Illumina + Hi-C	Illumina + PacBio
Genome size (Mb)	516.11	580.5	537.64
Raw data of short reads sequencing (Gb)	58.14	76.46	84
Raw data of PacBio sequencing (Gb)	39.55	/	10
Contig N50 (Kb)	491.30	4.73	58.87
Scaffold N50 (Mb)	16.24	13.64	0.25
Number of contigs	2802	418683	29670
Number of scaffolds	405	236802	13327
Hi-C anchored rate(%)	96.15	89.19	/
Number of protein-coding genes	17739	35725	18733
Average gene length (bp)	12489	5591	9587
Average exon per gene	6.99	4.68	6.54
Repeated sequences (%)	45.07	46.03	39

### Genome annotation

**Fig. 2:**
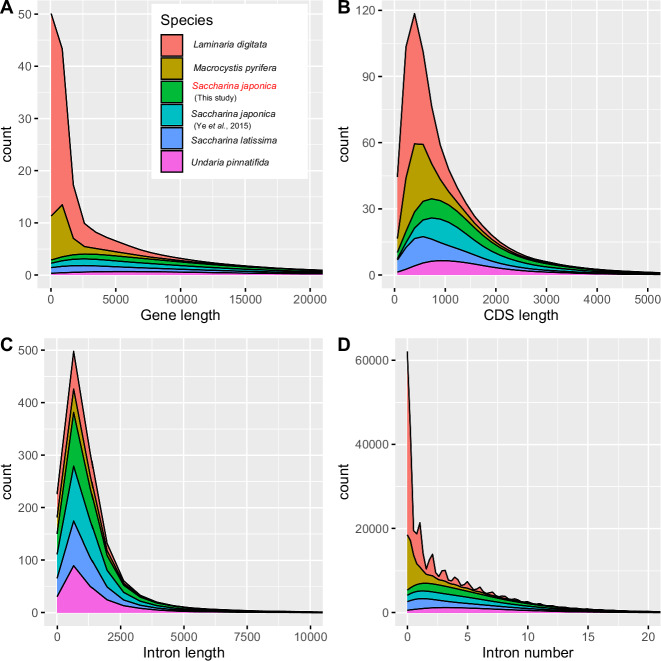
The composition of gene elements in *S. japonica* and other closely related species. (**A**) Distribution of gene length. (**B**) Distribution of CDS length. (**C**) Distribution of intron length. (**D**) Distribution of intron number.

Genes were predicted using evidence from both ab initio gene predictors and protein and transcript alignments. First, a de novo repeat library was constructed using EDTA v2.2.1^22^, and repeats were identified using RepeatMasker v4.1.2 (https://repeatmasker.org/RepeatMasker/). Repeat sequences were soft masked before further gene predictions. Secondly, Braker pipeline v3.0.3^23^ was used to train the gene prediction tools GeneMark-ETP^24^ and Augustus v3.5.0^25^ and to generate the ab initio predictions based on RNA-seq data and proteins from OrthoDB v11^26^. Thirdly, RNA-seq reads (The raw data is deposited in NCBI, with Bioproject accession of PRJNA1181304) were assembled using Trinity v2.15.1^27^, and the results were passed to PASApipeline v2.5.3^28^ to generate high quality gene structures. Fourth, evidence from protein sequences of OrthoDB v11 and RNA-seq reads was extracted from the alignments to the genome by using MetaEuk^29^, Minimap2 v2.26-r1175^30^ and StringTie v2.2.1^31^. Finally, the above ab initio gene predictions and protein and transcript alignments were combined into weighted consensus gene structures using EVidenceModeler v2.1.0^32^ implemented by Funannotate v1.8.17^33^ (https://github.com/nextgenusfs/funannotate). Protein functions were annotated using Funannotate from alignments on multiple databases including the Swiss-Prot/TrEMBL^34^, Pfam-A^35^, EggNOG^36^ and InterProScan^37^. A total of 17,739 protein-coding genes are predicted, of which 14,510 (82%) genes are functionally annotated. The statistics of gene models, including gene length, CDS length, intron length and intron length in *S. japonica* were comparable to other Laminariales algae (Fig. 2).

## Data Records

The sequencing data from Illumina has been deposited in Sequence Read Archive (SRA) database at NCBI (SRX26590917)^38^.

The sequencing data from PacBio has been deposited in Sequence Read Archive (SRA) database at NCBI (SRX26590916)^39^.

The sequencing data from Hi-C has been deposited in Sequence Read Archive (SRA) database at NCBI (SRX26590918)^40^.

The assembly genome data was deposited in GenBank at NCBI (JBIYRG000000000.1)^41^.

The genomic assembly and annotation results were deposited in the Figshare database (10.6084/m9.figshare.27569472)^42^.

## Technical Validation

The quality of the genome assembly was checked using a k-mer based method as implemented in Merqury v1.3^43^, resulting a QV score of 34.86 and completeness of 96.67%. We further mapped the paired-end short reads to the genome using bwa-mem2 v2.2.1^44^, resulting a mapping ratio of 87.16% and proper paired mapping ratio of 83.62%.

## Data Availability

No custom code was used in this study. The data analyses used standard bioinformatic tools specified in the methods.
